# M2 Macrophages are Major Mediators of Germline Risk of Endometriosis and Explain Pleiotropy With Comorbid Traits

**DOI:** 10.1002/advs.202415285

**Published:** 2025-09-12

**Authors:** Soledad Ochoa, Fernanda S. Rasquel‐Oliveira, Brett McKinnon, Marcela Haro, Sugarniya Subramaniam, Pak Yu, Simon Coetzee, Michael S. Anglesio, Kelly N. Wright, Raanan Meyer, Anne E. Porter, Caroline E. Gargett, Sally Mortlock, Grant W. Montgomery, Michael S. Rogers, Kate Lawrenson

**Affiliations:** ^1^ Department of Obstetrics and Gynecology University of Texas Health San Antonio TX 78229 USA; ^2^ Center for Inherited Oncogenesis University of Texas Health San Antonio TX 78229 USA; ^3^ Vascular Biology Program Boston Children's Hospital Department of Surgery, Harvard Medical School Boston MA 02115 USA; ^4^ The Institute for Molecular Bioscience The University of Queensland Brisbane Australia 4072; ^5^ Women's Cancer Research Program at the Samuel Oschin Comprehensive Cancer Center Cedars‐Sinai Medical Center Los Angeles CA 90048 USA; ^6^ British Columbia's Gynecological Cancer Research (OVCARE) Program University of British Columbia Vancouver General Hospital and BC Cancer Vancouver British Columbia V6T 1Z1 Canada; ^7^ Department of Obstetrics and Gynecology UBC Vancouver British Columbia V6T 1Z4 Canada; ^8^ Division of Minimally Invasive Gynecologic Surgery Department of Obstetrics and Gynecology Cedars‐Sinai Medical Center Los Angeles CA 90048 USA; ^9^ The Ritchie Center Hudson Institute for Medical Research Melbourne Victoria 3168 Australia; ^10^ Department of Obstetrics and Gynaecology Monash University Melbourne Victoria 3800 Australia

**Keywords:** anakinra, angiogenesis, endometriosis, GWAS, IL1A, IL1B, inflammation, M2 macrophages, organoids, single cell transcriptomics

## Abstract

Endometriosis is a common gynecologic condition that causes chronic, life‐altering symptoms including pain and infertility. There is an urgent need for new non‐hormonal targeted therapeutics to treat endometriosis, but until very recently, the cellular and molecular signatures of endometriotic lesions are undefined, hindering the development of clinical advances. Integrating inherited risk data from analyses of >45 0000 individuals with ≈35 0000 single‐cell transcriptomes from 21 patients, M2‐macrophages as candidate drivers of disease susceptibility are uncovered, and nominating IL1 signaling as a central hub impacted by germline genetic variation associated with endometriosis risk. Extensive functional follow‐up confirmed these associations and revealed a pleiotropic role for this pathway in endometriosis. Population‐scale expression quantitative trait locus analysis demonstrates that genetic variation controlling *IL1A* expression is associated with endometriosis risk variants. Manipulation of IL1 signaling in state‐of‐the‐art in vitro decidualized endometrial organoids impacts epithelial differentiation, and in an in vivo endometriosis model, treatment with anakinra (an interleukin‐1 receptor antagonist) results in a significant, dose‐dependent reduction in spontaneous and evoked pain and dampened pro‐angiogenic signaling. Together, these studies highlight non‐diagnostic cell types as central to endometriosis susceptibility and support IL1 signaling as an important actionable pathway for this disease.

## Graphical abstract

Genes associated with endometriosis risk are highly expressed in M2 macrophages, particularly ligands *IL1A* and *IL1B*. Endometrial‐type epithelium and stroma are likely recipients of these ligands. While *IL1B* treatment disturbs epithelial organization, blockage of the signaling results in a dose‐dependent decrease in spontanesous and evoked pain, reduces tissue burden, and decreases the concentration of angiogenesis regulators.

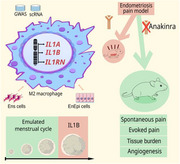

## 1 Introduction

Endometriosis is a common yet poorly understood gynecological disease, affecting around 10% of women and transgender men^[^ ^1^ ^]^ at reproductive‐age. In endometriosis, glands containing endometrial‐like epithelium and stroma are found outside of the uterine cavity, often within the ovaries and throughout the peritoneal cavity. Endometriosis causes chronic pain, dysmenorrhea, and infertility, and is associated with an increased risk of rare types of ovarian cancer.^[^ ^2^, ^3^ ^]^ While the presence of endometrial‐type epithelium and stroma is diagnostic for endometriosis, other microenvironmental cell types play essential roles in disease pathogenesis. Fibrosis and dysregulated innate and adaptive immune responses are other hallmarks of endometriosis; elucidating the pathways underpinning these processes is likely to highlight alternative therapeutic targets.^[^ ^4^, ^5^ ^]^ Genome‐wide association studies (GWAS) profile common genetic polymorphisms across population‐scale cohorts to identify germline variants associated with susceptibility to complex traits. GWAS have identified thousands of associations that impact risk for thousands of phenotypes, including endometriosis.^[^ ^6^, ^7^, ^8^, ^9^ ^]^ The era of GWAS spawned a large field of “post‐GWAS” functional projects aiming to construct the pathways by which risk variants, typically located in noncoding DNA, perturb gene expression to impact disease susceptibility.^[^ ^10^ ^]^ For endometriosis risk variants, a number of studies have quantified mRNA expression, miRNA expression, or methylation in eutopic endometrium to identify candidate genes, particularly those expressed in epithelial cells, associated with susceptibility to endometriosis.^[^ ^11^, ^12^, ^13^, ^14^ ^]^ Here we extend this work by integrating single‐cell landscapes of endometriosis to explore the role of the lesion microenvironment in endometriosis risk. Specifically, we are able to identify associated cell types and potential risk genes by evaluating the expression of GWAS‐prioritized genes in individual cells. We report a central role for myeloid cells in endometriosis risk that may also explain genetic overlaps with certain cancers, pain conditions, and inflammatory traits.

## 2 Results

### 2.1 Single Cell Disease Risk Scores Associate M2 Macrophages and Endometriosis Risk

**Figure 1:**
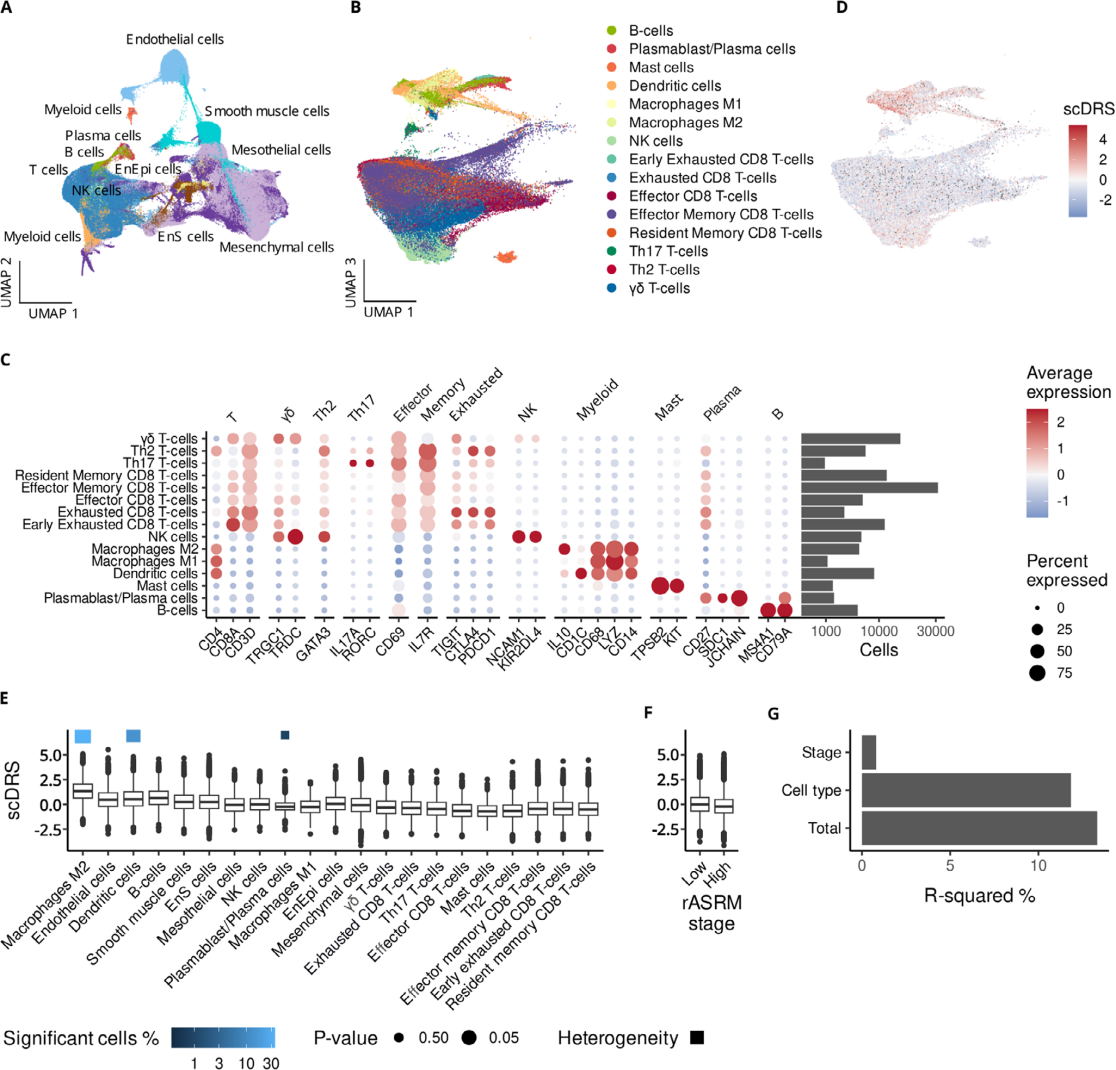
Endometriosis risk gene expression is enriched in M2 macrophages, dendritic cells and plasmablasts/plasma cells. A) UMAP representation of the major immune and structural cell types in endometriosis. The data set includes 362700 cells from 49 samples taken from 21 patients. Erythrocytes are excluded. B) UMAP representation of the immune subclusters present. C) Expression of the canonical markers that define the immune clusters. D–F) Distribution of the single cell disease relevance score in D) the UMAP space, E) per cell cluster, and F) stage. The Monte Carlo test is significant for M2 macrophages (*p* = 0.01), dendritic cells (*p* = 0.05), and endothelial cells (*p* = 0.04). Geary's C as a test statistic for heterogeneity within cell type is significant for macrophages (*p *= 0.03), dendritic cells (*p* = 0.03), and plasma cells/plasmablasts (*p* = 0.05). G) scDRS variance explained by different cell categories according to linear regression. UMAP, uniform manifold approximation and projection; NK, natural killer cells. Only the 200798 cells from endometrioma and peritoneal endometriosis are included in F). Each stage group is represented by 7 patients. In E) and F) upper and lower bounds of boxes represent upper and lower quartiles, with central lines at the median. Solid lines above and below the boxes denote the minimum and maximum data points, and black dots denote outliers. Blue squares highlight cell populations with significant heterogeneity in risk scores (*p *< 0.05).

Given the known importance of immunity and inflammation in endometriosis, we annotated immune subsets in reanalyzed data from a single cell atlas of peritoneal endometriosis (*n* = 32 samples), endometrioma (*n* = 8 samples), endometriosis‐free ovaries (*n* = 4 samples), and eutopic endometrium (*n* = 10 samples) from 21 patients.^[^ ^15^ ^]^ 118103 CD45‐positive (*PTPRC*‐expressing) immune cells were stratified into 15 functionally distinct populations (see Experimental Section and Table S1, Supporting Information). T‐cell subsets include 5 clusters of CD8 T‐cells (63906 cells), gamma‐delta T‐cells (γδ; 16284 cells), Th2 CD4 T‐cells (6843 cells), and Th17 CD4 T‐cells (920 cells). Myeloid subsets include 8790 dendritic cells, 1139 M1 macrophages, and 5554 M2 macrophages. The remaining immune subsets comprise B cells (5274 cells), plasmablast/plasma cells (1771 cells), natural killer (NK) cells (5977 cells), and mast cells (1645 cells).^[^ ^15^, ^16^ ^]^ Other cell types present in the data set include the endometrial‐type epithelium (8160 cells) and endometrial‐type stroma (66721 cells) that comprise the glandular structures seen in eutopic endometrium and endometriosis, plus mesenchymal cells (125311 cells), mesothelial cells (2865 cells), and endothelial cells (23226 cells) (**Figure** **1**A–C).

The single cell disease relevance score (scDRS)^[^ ^17^ ^]^ software was used to identify cell types/subtypes associated with endometriosis risk by integrating the single cell transcriptome profiles with genome‐wide association data from a meta‐analysis of 450668 controls and 23492 endometriosis cases.^[^ ^6^ ^]^ This association study included patients with both minimal/mild (revised American Society for Reproductive Medicine (rASRM) stage I/II) and severe/extensive (rASRM stage III/IV); most subjects were of European ancestry. The scDRS algorithm starts by calculating the score per cell and then tests distributions for cell type‐level association and heterogeneity. Among immune cells, the highest scores were observed in the myeloid cluster, with T‐cells tending toward negative values, and NK cells showing a slight increase (Figure 1D). The top five cell types by average score are M2 macrophages (1.38), B cells (0.68), dendritic cells (0.60), endothelial cells (0.49), and smooth muscle cells (0.27). A significant association with inherited risk of endometriosis was found for M2 macrophages (*p* = 0.01), dendritic cells (*p* = 0.05), and endothelial cells (*p* = 0.04), with B cells not reaching statistical significance (Monte Carlo test *p* = 0.051). Within cell‐type heterogeneity was detected for M2 macrophages (*p* = 0.03), dendritic cells (*p* = 0.03), and plasma cells/plasmablasts (*p* = 0.05), indicating subsets of these cells drive association with disease (Figure 1E; Table S2, Supporting information). Although high‐stage endometriosis has greater heritability,^[^ ^18^ ^]^ when stratifying cells based on stages, we did not see a difference in scDRS score for cells isolated from patients with stage I/II compared to stage III/IV disease (Figure 1F). Cell type explained 14.65 times more variance in score compared to stage (Figure 1G).

### 2.2 Macrophage‐Derived *IL1A/IL1B* as Likely Mediators of Risk Conferred by Genetic Variants at 2q41.1

**Figure 2:**
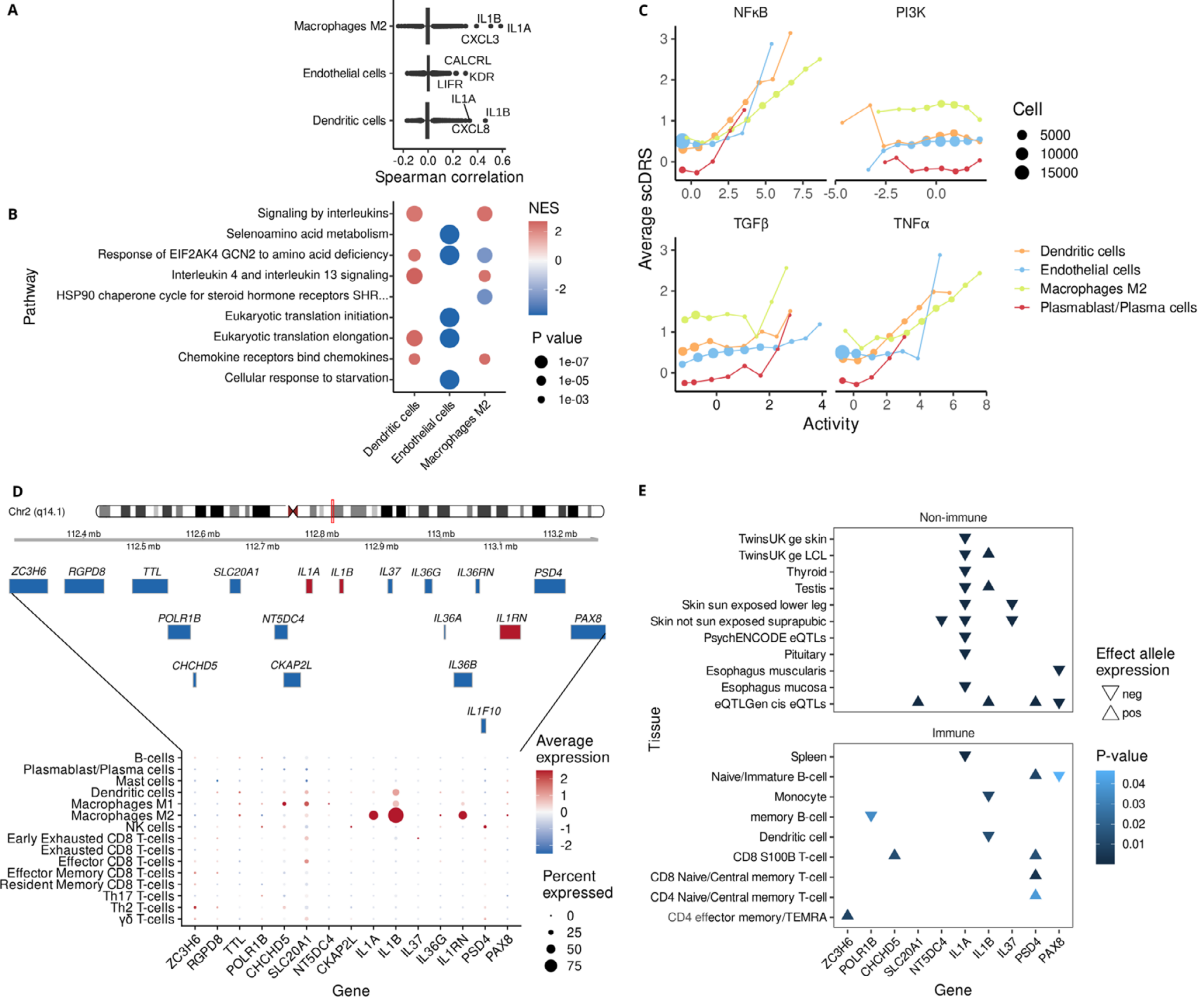
Endometriosis risk is consistently associated with *IL1A/IL1B* gene expression. A) Spearman correlation between gene expression and scDRS. B) Top five enriched pathways among genes correlated with scDRS. C) scDRS heterogeneity relative to PROGENy pathway activity. Spearman correlation. D) Gene expression of candidate susceptibility genes at the 2q41.1 endometriosis risk locus in immune subsets. E) Expression quantitative trait locus analysis of differences in candidate risk gene expression associated with endometriosis risk variant genotype.

To better understand the association with M2 macrophages, dendritic cells, and endothelial cells, we identified the genes with expression correlated with the risk score (**Figure** **2**A), and the pathways they affect (Figure 2B). Correlation values were centered around 0 for the 30354 features in the dataset, but 1642, 3365, and 4157 genes were significantly correlated (adjusted *p* < 0.05) with the scDRS score in M2 macrophages, dendritic cells, and endothelial cells, respectively. *IL1A* and *IL1B* were the top‐ranked genes with the highest positive correlation in both myeloid subgroups (*IL1A* Spearman correlation 0.59 for M2 macrophages, 0.36 for dendritic cells; *IL1B* correlation 0.51 for M2 macrophages and 0.46 for dendritic cells, adjusted *p* < 2.61 × 10^−222^ for each test). The top genes for endothelial cells were distinct but included genes known to be involved in endometriosis. Variants upstream of *KDR*, encoding VEGFR2, have been associated with disease severity; CALCRL functions as co‐receptor of CGRP, which affects lesion size and pain,^[^ ^19^ ^]^ and, finally, the LIF receptor seems to contribute to lesion immunomodulation.^[^ ^20^, ^21^ ^]^ (Figure 2A). At the pathway level, signaling by different interleukins was highly enriched among the positively correlated genes for myeloid cells (adjusted *p* < 1.97 × 10^−08^) (Figure 2B). Additionally, translation‐related pathways were highly represented, particularly among negatively correlated genes for endothelial cells.

Of the three cell types associated with endometriosis, both myeloid populations displayed significant within‐cell‐type disease‐association heterogeneity (Figure 1E). To check if this heterogeneity is associated with cell function, we estimated pathway activity using PROGENy scores.^[^ ^22^ ^]^ Comparing binned pathway scores against average scDRS, a positive correlation between risk and pathway activity was evident for NF‐κB (Spearman correlation = 0.92, *p*‐value 2.01 × 10^−7^) and TNF‐α (Spearman correlation = 0.80, *p* 1.2 × 10^−6^), with M2 macrophages reaching higher levels of activity than dendritic cells, plasma cells or endothelial cells (Figure 2C). No other significant correlations between scDRS bin and pathway activity were detected, including for PI3K, a pathway somatically altered in endometriosis^[^ ^23^ ^]^ (Figure 2C; Figure S1, Supporting Information). IL1A and IL1B function in a feedback loop with NFκB signaling^[^ ^24^ ^]^ and have been proposed as the likely causal risk genes at chromosome 2q14.1, a locus associated with endometriosis across multiple populations.^[^ ^25^, ^26^, ^27^ ^]^ Endometriosis risk variants lie around 2 kb centromeric to the promoter of *interleukin 1A*. While the locus harbors over a dozen protein‐coding genes, most candidate risk genes were not expressed in any of the cell types present in the single‐cell endometriosis atlas (Figure 2D). *IL1A* and *IL1B* were notable for high expression in M2 macrophages. Endometriosis‐associated M2 macrophages also express IL1A/B antagonist *IL1RN*, which may also contribute to fine‐tuning of IL1 signaling and risk conferred by variants at this locus. Together, these data suggest that M2 macrophage‐derived IL1A/B could be involved in endometriosis risk.

### 2.3 Expression Quantitative Trait Locus (eQTL) Analysis Links Endometriosis Risk SNPs to the Expression of Candidate Genes Including *IL1A* and *IL1B*

Endometriosis risk variants in the chromosome 2q14.1 locus were mapped to eQTLs to identify evidence that genetically regulated expression differences in *IL1A* and/or *IL1B* are conferred by the same variants associated with endometriosis risk. Using the Fuma software and One1K single cell eQTL data, we interrogated 139 eQTL datasets representing 37 different tissue types (Table S3, Supporting Information). Decreased expression of *IL1A* was associated with increased risk in 10 different tissue types (*p < *10^−5^). Elevated endometriosis risk was associated with lower expression of *IL1B* in two myeloid cell types (monocytes, dendritic cells) and elevated expression in three data sets (Figure 2E). The greatest number of associations was detected for *IL1A* (10 eQTLs). Five eQTL associations were detected for *IL1B* and *PSD4*. The number and strength of associations for *IL1A*, and the myeloid‐specific associations for *IL1B* are further evidence to link these genes to endometriosis risk. To test whether the same variants are associated with risk and *IL1A* and/or *IL1B* expression, colocalization analyses were performed on a selection of tissue types. This revealed evidence of a shared causal variant between the endometriosis risk variants and a *IL1A* eQTL (Table S4, Supporting Information). The signals for *IL1B* eQTLs look to be independent of the endometriosis risk association and are likely a consequence of linkage disequilibrium.

### 2.4 M2 Macrophages are Central to Paracrine Signaling in Endometriosis

**Figure 3:**
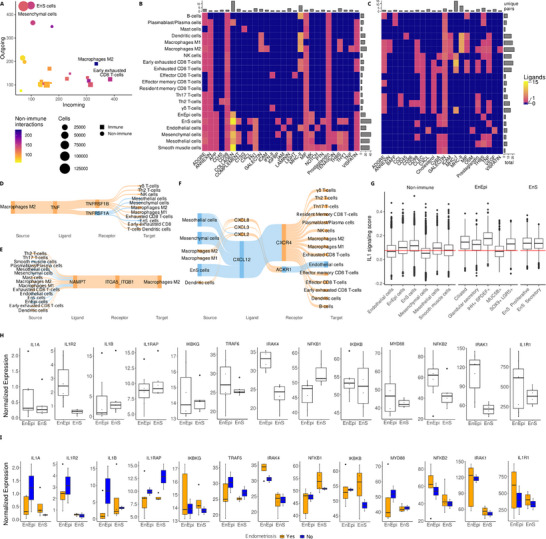
M2 macrophages play a central role in cell‐cell communications in endometriosis. A) Predicted interactions for every cell cluster. B,C) pathways affected by B) incoming and, C) outgoing signals for M2 macrophages. D–F) Ligand‐receptor pairs identified for D) TNF, E) Visfatin, F) CXCL. G) IL1 signaling expression in non‐immune cell clusters and subclusters. H) Expression of interleukin‐1 pathway genes in EnEpi and EnS. I) Expression of interleukin‐1 pathway genes in EnEpi and EnS of eutopic endometrium from patients with and without a confirmed diagnosis of endometriosis. Genes are sorted by expression rank. EnEpi, endometrial‐type epithelium; EnS, endometrial‐type stroma; NK, natural killer cells. In H and I genes are ordered from low to high expression.

CellChat^[^ ^28^ ^]^ was used to predict signaling between cell types in endometriosis. In total 192 ligand‐receptor pairs and 4056 predicted connections between cell types were identified (*p *< 0.05, Table S5, Supporting Information), since different cell types can communicate through the same ligand‐receptor pairs. Non‐immune cells were predicted to form more outgoing than incoming links, while the opposite happened for immune cells (**Figure** **3**A). For example, endometrial‐type stromal cells exhibit 439 outgoing and 99 incoming connections, by contrast, early exhausted T‐cells are involved in 124 outgoing and 385 incoming signals. Non‐immune cells tended to be more connected than immune cells, with an average of 465 and 362 interactions, respectively. Among immune cells, M2 macrophages and early exhausted T‐cells are particularly connected, with 549 and 528 connections, each. Compared to other immune cells, M2 macrophages were exceptionally connected with non‐immune cells, which form 229 interactions with M2 macrophages, contrasting to 157 connections for early exhausted T‐cells, the second most connected subset.

Interactions between non‐immune cells and M2 macrophages were mostly incoming and concentrated on the collagen pathway (Figure 3B,C). There were pathways affected by the same small set of ligand‐receptor pairs expressed indiscriminately across various cell types, like incoming CD99 and outgoing galectin, and pathways linked with distinct ligand‐receptor pairs affected in a small set of cell types, like incoming collagen, incoming MHC‐I and both incoming and outgoing MHC‐II. Non‐immune cells were under‐represented among the recipients of outgoing interactions from M2 macrophages, with myeloid cells, exhausted CD8 T‐cells, and NK cells being the predominant targets. All this suggests that M2 macrophages play a central role in the cross‐talk between non‐immune clusters used for diagnosis and the immune groups. Overall, M2 macrophages represented a communication bottleneck for several pathways, including outgoing TNF (Figure 3D) and incoming visfatin^[^ ^29^ ^]^ signaling (Figure 3E). In other instances, such as the CXCL pathway, myeloid cells, including M2 macrophages, participated in incoming and outgoing signaling with a variety of cell types, with specificity determined by the receptor and ligand molecules expressed (Figure 3F)

IL1 signaling was not identified in the CellChat analyses, likely due to low expression of the coreceptor *IL1RAP*. We therefore performed a targeted survey of IL1 signaling among the non‐immune cell types, searching for possible effector cells for the IL1A/B produced by M2 macrophages. Except MUC5+ endometrial‐type epithelial cells, all endometrial‐type epithelial (EnEpi) and stromal (EnS) subtypes surpassed the mean expression of an IL1 transcriptional signature among non‐immune cell types, indicating that they are likely recipients of IL1 signaling. Endometrial‐type stromal cells and the IHH+SPDEF+ subset of endometrial‐type epithelial cells exhibited the highest average expression of the IL1 signature among non‐immune cell types (Figure 3G; Figure S2, Supporting Information). To evaluate the expression of IL1 signaling genes in cells from the eutopic endometrium from cases and controls, bulk‐RNA sequencing data were generated from freshly isolated primary EnEpi and EnS. Bulk RNA‐sequencing provides more depth and can identify more lowly expressed genes compared to single‐cell sequencing. Analysis of 13 genes involved in IL1 signaling revealed similar patterns of expression in epithelial and stromal cells, with more interpatient variation within EnEpi (Figure 3H). IL1R2 (*p *< 0.01) and signaling intermediaries IRAK1 (adjusted *p *= 0.02) and IRAK4 (adjusted *p *= 0.01) show the highest significant difference between EnEpi and EnS. IL1R1 has high expression in both cell types, but the associated protein IL1RAP is relatively low, consistent with the single‐cell data. IL1A and IL1B were lowly expressed relative to receptor IL1R1 and effectors IRAK1 and NFKB2, consistent with the epithelium and stroma being receivers of IL1 signaling but not major sources of these interleukins. There was also a tendency for eutopic endometrium from endometriosis cases to express higher IL1R1 and lower IL1A and IL1B (Figure 3I).

### 2.5 IL1B Treatment of Endometrial Organoid Co‐Cultures in vitro Disrupts Epithelial Differentiation

**Figure 4:**
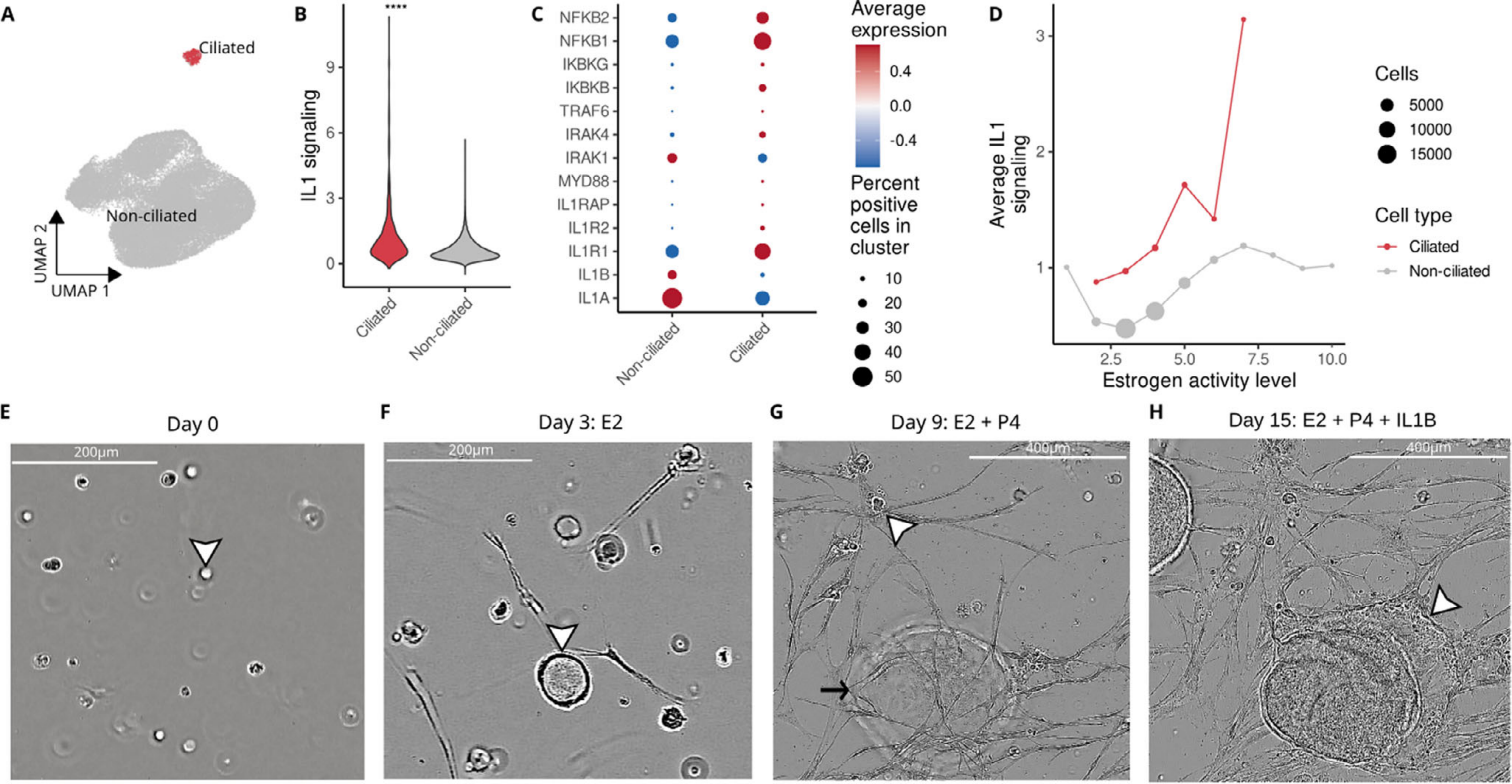
Stimulation of interleukin‐1 signaling disrupts the organization of human endometrial organoid‐stromal co‐cultures. A) UMAP representation of single‐cell transcriptomes for 39568 endometrial epithelial cells from organoids. B) IL1 signaling for ciliated and non‐ciliated cells. T‐test *p* < 0.0001(****). C) Expression of selected markers from IL1 signaling pathway. D) Spearman correlation between estrogen activity and IL1 signaling (*p* = 0.017). E‐G) 3‐D Incucyte photos capturing the development of the organoid co‐cultures. E) Single cell seeding of epithelial and mesenchymal stem cells. Arrowhead denotes a representative single cell. F) Treatment with estradiol (E2) and cell proliferation. Arrowhead shows a representative proliferating organoid. G) Treatment with E2 in combination with progesterone (P4), stromal cell decidualization (arrowhead), and epithelial organoid expansion (arrow). H) Treatment with IL1B results in disordered epithelial differentiation (arrowhead).

When maintained in Matrigel with defined growth factors, EnEpi cells co‐cultured with EnS or endometrial mesenchymal stem cells spontaneously create complex organotypic structures that closely mimic the histology and functional differentiation of human endometrial tissues in vivo.^[^ ^30^ ^]^ Epithelial cells were isolated from menstrual fluid from six patients with suspected endometriosis, five had endometriosis confirmed through laparoscopic surgery, and the sixth did not undergo surgery, leaving endometriosis status unconfirmed. Stromal cells were isolated from endometrial biopsies from 8 different patients (4 with endometriosis and 4 without endometriosis), and the two cell types were co‐cultured to establish organoid‐stromal co‐cultures (see Methods). Phenotypic details of each patient are included in Table S6 (Supporting Information). After single‐cell RNA‐sequencing of the organoids, cell identities were transferred from the endometriosis cell atlas, stratifying epithelial cells into ciliated and secretory subsets (**Figure** **4**A; Figure S3, Supporting Information). IL1 signaling was elevated in ciliated cells relative to non‐ciliated epithelia, although IL1A and IL1B were lowly expressed (Figure 4B,C). IL1 signaling was positively correlated with estrogen signaling in ciliated cells only (Figure 4D and *p *= 0.017), consistent with the known role of estrogen on ciliogenesis^[^ ^31^ ^]^ and the substantial overlap in the transcriptional targets of estrogen and IL1 (89.47% of the IL1 signaling genes are also estrogen regulated).^[^ ^32^ ^]^

To test the impact of IL1B exposure in vitro, single cell suspensions of endometrial epithelial cells and endometrial mesenchymal stem cells were seeded into Matrigel and cultured under conditions to facilitate the generation of 3D in vitro endometrial organoids (Figure 4E). To mimic endometrial development and changes across the menstrual cycle, organoid‐stromal co‐cultures were treated with estradiol (E2), which stimulated cell proliferation, characteristic of proliferative (follicular) phase endometrium (Figure 4F). A combination of E2 with progesterone (P4) resulted in a transition to elongated decidualized mesenchymal stromal cell knots and expansion of epithelial glandular structures (Figure 4G), which mimic the morphology and structure of mature secretory endometrium. Treatment of mature, decidualized organoid‐stromal co‐cultures with IL1B resulted in a drastic alteration in cellular morphology, with extensive reorganization of the epithelial glandular structures. Disrupted glandular differentiation resulted in epithelial spread and infiltration into the surrounding decidualized mesenchyme (Figure 4H; Video S1, Supporting Information)

### 2.6 IL1R Antagonist Therapy Reduces Pain Sensitivity in vivo

**Figure 5:**
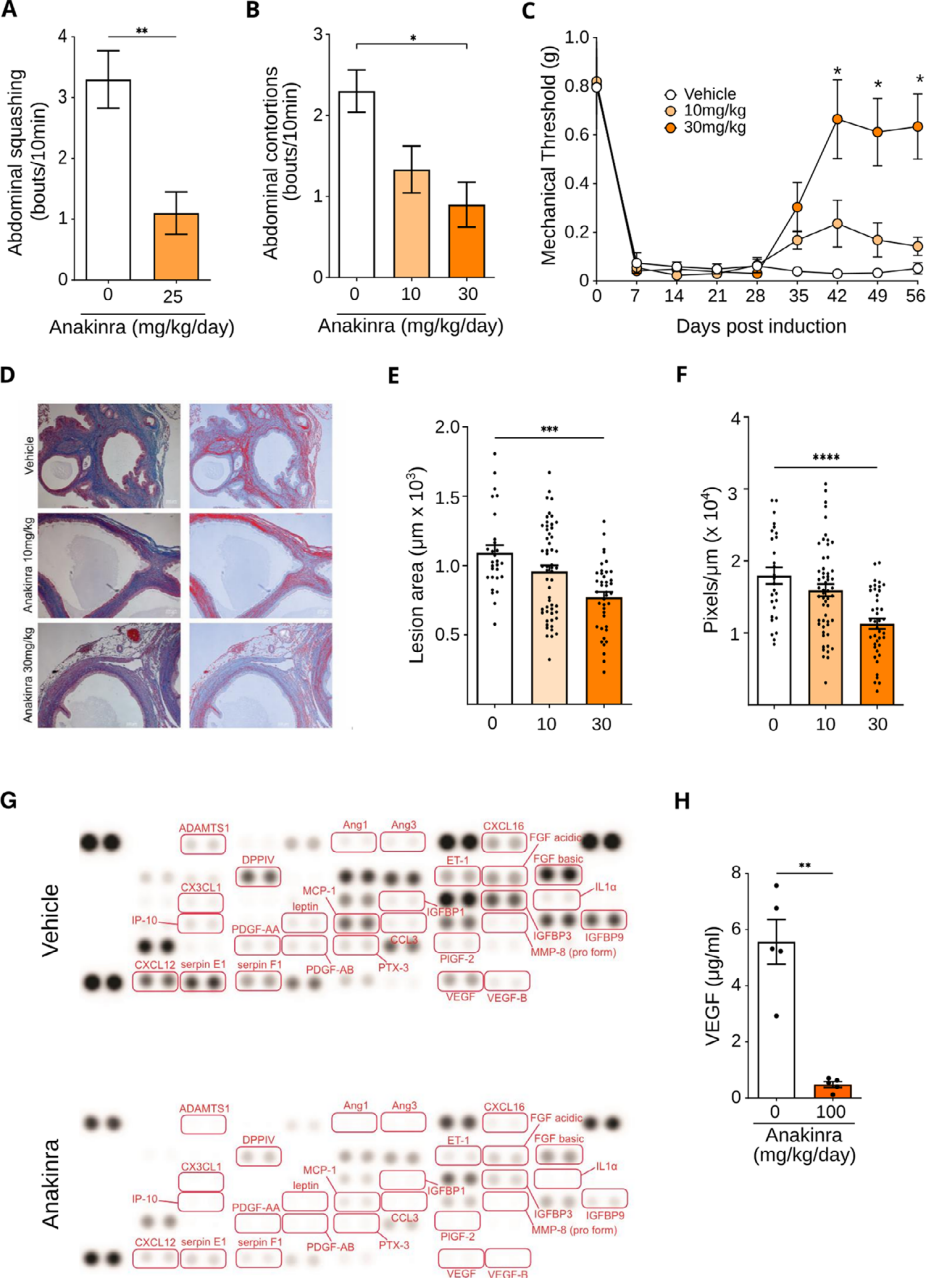
Blocking IL1 signaling reduces endometriosis‐associated pain in a mouse model. Endometriosis‐like lesions were induced in C57BL/6J mice, allowed to grow for 4 weeks, and treated with anakinra at the indicated dose. Spontaneous pain was decreased after treatment as measured by A) Abdominal squashing and B) abdominal contortions. C) Evoked pain measured using von Frey fibers on the abdomen was also decreased in a dose‐dependent manner. D) Masson's trichrome staining of the resulting lesions, including representative microscopic images (left) and masks (right) indicating in red pixels scored as positive for collagen staining (blue). E) Lesion area measured and F) area stained positive for collagen. G) Angiogenesis dot‐blot array using extracts from vehicle and anakinra‐treated lesions. Highlighted growth factors showed at least two‐fold reduction in mean normalized signal. H) ELISA‐measured VEGF concentration in vehicle‐ and anakinra‐treated lesions. ^*^ *p* < 0.05, ^**^ *p* < 0.01, ^*****^ *p* < 0.001, ^****^ *p* < 0.0001 by ANOVA (B,C,E,F) or Student's *t*‐test (A,H). Investigators were blinded to group allocation until all analyses were complete. *n* = 10 mice per group, except for the 10 mg kg^−1^ group, where *n* = 9.

Since the scDRS and in vitro results support a central role for IL1 signaling in endometriosis pathogenesis, we evaluated the effect of using anakinra to disrupt intercellular IL1 signaling in a validated mouse model of endometriosis‐associated pain.^[^ ^33^ ^]^ Anakinra is a pharmacologically optimized recombinant version of interleukin‐1 receptor antagonist, the product of the *IL1RN* gene. Endometriosis‐like lesions were induced in mice by injecting dissociated uterine fragments from estrogen‐primed donor animals into the peritoneal cavity of syngeneic recipients. A blinded investigator measured evoked pain using von Frey fibers on day 0 and weekly thereafter. On day 29, animals were block randomized to treatment groups and treated for 4 weeks. At the end of the experiment, a blinded investigator measured evoked pain, spontaneous pain (abdominal squashing and abdominal contortions), and lesion size and number. Anakinra treatment results in a significant, dose‐dependent decrease in spontaneous pain and evoked pain (**Figure** **5**A–C; Figure S4, Supporting Information).

Lesions were then sectioned and stained using Masson's trichrome and evaluated for tissue area and collagen staining. We found that anakinra treatment reduced tissue burden (**Figure** **5**D,E), even though gross lesion size was not different among groups. Similarly, we observed a decrease in lesion collagen staining (Figure 5F). As IL1 signaling is known to substantially affect angiogenesis, we also evaluated the effect of anakinra treatment on the angiogenic regulatory milieu in lesion lysates. As measured by dot‐blot, we found that treatment decreased the concentration of over 20 angiogenesis regulators by at least two‐fold (Figure 5G). Most prominently, it decreased the concentration of VEGF in lesions by over 10‐fold (Figure 5H, vehicle, 5.6 µg mL^−1^ vs. anakinra, 0.5 µg mL^−1^). Together, these data indicate that IL1 contributes to lesion progression by promoting angiogenesis, increasing tissue burden, and fibrosis.

### 2.7 M2 Macrophages Explain Genetic Interactions With Immune and Pain Conditions

**Figure 6:**
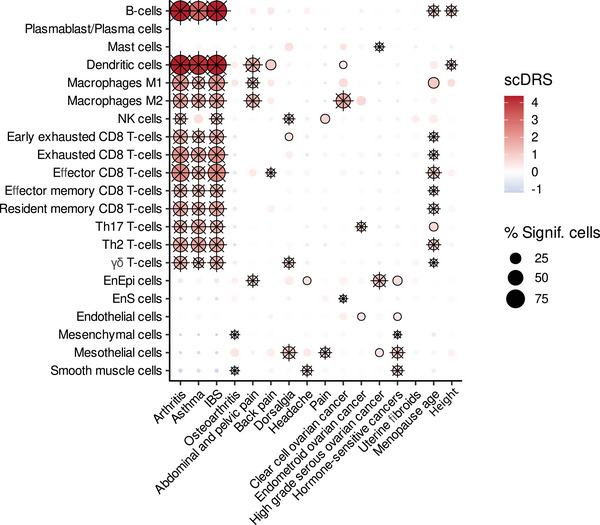
scDRS identifies cell types that may explain associations with traits that have shared germline genetics with endometriosis. scDRS scores for other traits that share risk genetics with endometriosis. A black border highlights cell types with significant associations with the trait. An asterisk indicates significant heterogeneity detected.

An extensive body of literature documents epidemiologic and genetic links between endometriosis and inflammatory conditions. Asthma (odds ratio^[^OR] = 1.35, 95% confidence interval [CI] = 0.97–1.88), chronic fatigue syndrome and/or fibromyalgia (OR = 5.81, 95% CI = 1.89–17.9), mononucleosis (OR = 1.75, 95% CI = 1.14–2.68), allergy (OR = 1.76, 95% CI = 1.32–2.36) and rheumatoid arthritis (incidence rate ratio = 1.31, 95% CI: 1.05–1.64) are all associated with endometriosis.^[^ ^6^, ^34^, ^35^, ^36^, ^37^, ^38^ ^]^ We have recently also identified a significant genetic correlation and causal relationship between genetic liability to inflammatory gastrointestinal disorders and endometriosis risk, and evidence for a bidirectional causal relationship between endometriosis and irritable bowel syndrome.^[^ ^39^ ^]^ To check for other risk associations of the endometriosis microenvironment, summary statistics from GWAS for 16 traits (Table S7, Supporting Information) were used to calculate the scDRS (Table S8, Supporting Information). B cells (but not plasma cells), dendritic cells, M1 and M2 macrophages as well as functional T‐cell subsets, were all associated with arthritis, asthma, and irritable bowel syndrome (**Figure** **6**). Dendritic cells, M1 and M2 macrophages, and endometrial‐type epithelium were all associated with abdominal and pelvic pain but not back pain. Endometriosis is associated with risk of clear cell and endometrioid ovarian cancer, but links with the high‐grade serous subtype are less consistent.^[^ ^2^, ^40^ ^]^ M2 macrophages and endometrial‐type stroma were associated with clear cell ovarian cancer (Figure 6). Endometrial‐type epithelium, mesothelial cells, and other structural cells had associations with high‐grade serous ovarian cancers and/or hormone‐sensitive cancers (postmenopausal breast, uterine, ovary, prostate, and thyroid), likely reflecting a shared cell‐intrinsic component to risk for these cancers. Somewhat unexpectedly, cell types associated with age at menopause were similar to those associated with arthritis, asthma, and irritable bowel syndrome, suggesting immune cells play a greater role in the inherited component of age at menopause than previously thought.

## 3 Discussion

Endometriosis has myriad long‐term negative effects on women's health. There are no known highly penetrant germline mutations that contribute to endometriosis risk; but a substantial component of endometriosis risk is inherited, with heritability estimated at 47%–51% from twin studies.^[^ ^41^, ^42^ ^]^ Most of the inherited risk for endometriosis is due to the combinatorial effects of common mild‐risk variants. So far, 42 risk loci have been identified for endometriosis.^[^ ^6^, ^7^, ^9^, ^43^, ^44^ ^]^ Interpretation of post‐GWAS studies to date has focused on the role of endometrial‐type epithelial cells, however, given the central role of the microenvironment in disease pathogenesis, this likely overlooks a critical aspect of risk. Here, by integrating single‐cell gene expression profiles of endometriosis with GWAS data for endometriosis, M2 macrophages, dendritic cells, and endothelial cells were associated with endometriosis risk, suggesting these cells play a role in disease susceptibility and development. Evidence for genetic variants proximal to the interleukin 1A gene locus was among the earliest risk regions identified for endometriosis.^[^ ^26^, ^27^ ^]^ Of all the cytokine families, IL1 is most associated with damaging inflammation^[^ ^45^, ^46^, ^47^ ^]^ and has been predicted to modulate endometriosis symptomatology.^[^ ^48^ ^]^ In this study, IL1A/IL1B signaling was identified as a major effector driving the association between M2 macrophages and endometriosis. Somewhat unexpectedly, in eQTL analyses, lower IL1A expression was associated with increased risk, suggesting that muted inflammatory responses may be protective against developing endometriosis. However, the eQTL results should be interpreted with caution as IL1A and IL1B are known to have highly context‐specific effects, and their roles in endometriosis development and symptomatology are not yet completely defined.^[^ ^49^ ^]^

Computational inference of cell‐cell communications defines M2 macrophages as a major hub of signaling between immune and non‐immune cells, with IL1 signatures activated in endometrial‐type stroma and epithelial subsets, identifying these diagnostic cells within lesions as effectors of IL1 signaling in endometriosis. IL1A and IL1B both signal through the interleukin receptor (*IL1R1*) to regulate NF‐κB, cJUN, and p38 MAPK signaling, inducing the expression of a host of genes, including IL8.^[^ ^50^ ^]^ Expression of IL8 (encoded by the *CXCL8* gene) was also associated with endometriosis single‐cell disease risk scores, suggesting multiple risk variants may converge to modulate different components of the IL1 pathway. Risk scores were positively correlated with NF‐κB and TNF‐α signaling, consistent with previous observations of a triggered inflammatory response in endometriosis macrophages.^[^ ^51^ ^]^ Expression QTL analyses identified associations with IL1A and IL1B in the blood, suggesting a model whereby macrophages, differentiated from monocytes that circulate in the blood, contribute to the risk of multiple inflammatory traits, including endometriosis.^[^ ^19^ ^]^ In this pleiotropic model, receptor expression occurs in a disease‐relevant cell type—here, the epithelial and stromal cells in endometriosis.^[^ ^52^ ^]^ Since IL1A is expressed in a constitutive manner while IL1B needs to be triggered, IL1A would be released first, facilitating seeding. Subsequent expression of IL1B would amplify the inflammatory signal and favor lesion growth.^[^ ^53^ ^]^ The stress of lesion establishment and subsequent inflammation would reinforce the positive feedback loop that maintains elevated IL1 signaling.^[^ ^52^ ^]^ If there is any similarity with endometrial hyperplasia, this epithelial‐macrophage interaction will be key for lesion proliferation.^[^ ^54^ ^]^

M2 macrophages and dendritic cells were also associated with immune‐related traits that have genetic associations with endometriosis,^[^ ^6^, ^55^ ^]^ indicating that pleiotropic multi‐cellular mechanisms may explain genetic overlap between pain and inflammatory conditions. The increase in proinflammatory macrophages seen in endometriosis patients 4 is reminiscent of the elevated ratio of M1/M2 macrophages occurring in autoimmunity, in opposition to expanded M2 populations associated with allergic asthma and tumor proliferation and invasion.^[^ ^56^ ^]^ IL1 signaling could feasibly be involved in the pleiotropic associations observed. Enhanced secretion of IL‐1β is a feature of both endometriosis and autoinflammatory diseases.^[^ ^57^ ^]^ Furthermore IL1R1 variants may link endometriosis and asthma, since both endometriosis and lung alveolar epithelial cells express this gene.^[^ ^58^ ^]^

In vivo targeting of IL1 signaling with anakinra reduced pain symptoms in mice with induced endometriosis. This is consistent with a recent pilot trial in humans, which reported reduced dysmenorrhea (pelvic pain with menstruation) and improved quality of life in patients receiving 3 months of treatment compared to placebo.^[^ ^59^ ^]^ Even though pain relief is often independent of lesion relief,^[^ ^18^, ^60^ ^]^ the decrease in lesion tissue content strongly suggests a reduction of tissue‐derived noxious agent(s) may explain the effect on pain. The link of IL1B protein expression with neurogenesis and with stromal cell migration and invasion,^[^ ^46^, ^61^ ^]^ suggests IL1 receptor antagonist treatment could have pleiotropic effects on lesions, rendering this a particularly attractive therapeutic strategy for endometriosis. The specific effect of anakinra on pain in animal studies is also promising, with administration of 10 mg/kg preventing complex regional pain syndrome,^[^ ^62^ ^]^ 30 mg/kg inhibiting osteosarcoma‐induced thermal hyperalgesia and 100 mg/kg blocking osteosarcoma‐induced mechanical hyperalgesia but requiring up to 300 mg/kg to prevent inflammatory thermal hyperalgesia.^[^ ^63^ ^]^ The moderate dose (30 mg kg^−1^) used here suggests efficacy, increasing the appeal of anakinra for endometriosis treatment. On the other hand, the interplay of IL1A and IL1B with IL18, IL33, IL36A, IL36B, IL36G, IL37, and IL38 indicate resistance through compensatory upregulation of intersecting pathways may be a challenge,^[^ ^64^ ^]^ so combination therapies could be considered. Then, even though assessment of IL‐1α levels in cervico‐vaginal fluid has potential for patient stratification,^[^ ^60^, ^65^ ^]^ given the complex nature of endometriosis‐associated inflammation, it is likely that other inflammatory markers also need to be accounted for.

A main limitation of this study is the dependency on gene expression. The single‐cell disease score evaluates excess expression of GWAS putative genes, but risk variants that reduce gene expression are overlooked when we focus on the top genes correlated with the score. The sample size of the gene expression dataset could also bias observations. Epigenome and functional studies will be needed to causally link endometriosis‐associated non‐coding genetic variation to M2 macrophage function and modified IL1 signaling. While treatment of epithelial‐stromal co‐cultures with recombinant IL1B minimizes confounding factors, we ultimately aim to integrate macrophages into these models; either isolating macrophages from individuals with relevant genotypes or engineering them using CRISPR/Cas9 genome editing. This will require extensive optimization of a triple co‐culture system, and large replicate numbers to account for genetic background variability.

Aside from surgery, hormonal therapy and pain management are the most common treatments for endometriosis. Hormonal therapy includes second‐line GnRH analogs, and first‐line progestins or combined hormonal contraceptives; which relieve pain by inhibiting the cyclic proliferation of endometriosis tissue.^[^ ^64^ ^]^ Even though response rates are fairly good, secondary effects may become problematic due to the extended periods of treatment needed for a condition, where about 50% of patients experience recurrence of symptoms.^[^ ^65^ ^]^ Relugolix and elagolix are the only new medications approved by the FDA for the treatment of endometriosis in the last 10 years, both of which are GnRh‐antagonists that produce a transient medical menopause and suppress ovulation. They reduce both dysmenorrhea and nonmenstrual pelvic pain at rates around 73% and 56%, with minimal loss of bone density when administered in combination therapy.^[^ ^66^, ^67^ ^]^ Due to side effects of hypoestrogenic state, these medications can only be used for 6–24 months, limiting their long‐term use. Among progestins, dienogest has been highly used; it is reported to provide equivalent to GnRH pain relief, and injury reduction, with side effects comprising menstrual alterations, osteopenia, headaches, weight gain, and libido reduction.^[^ ^67^ ^]^ Studies comparing dienogest with combined contraceptives find no difference in pain reduction, but more adverse effects on the combined contraceptives group, comprising abnormal uterine bleeding, mood swings, headache, nausea, and breast tenderness.^[^ ^67^, ^68^ ^]^ Beside the moderate to more severe side effects, hormonal therapy is not an option for patients planning pregnancy.

On the other hand, anakinra has been approved for the treatment of rheumatoid arthritis since 2001, and is being successfully used off‐label for the treatment of several inflammatory‐based diseases.^[^ ^69^ ^]^ Injection site reaction is the most common adverse effect, reported in 71% of patients. Though IL1 inhibitors have been linked to increased risk of serious infections, there is a dose effect specifically warning against high doses (⩾100 mg of anakinra).^[^ ^70^ ^]^ While no increase in adverse outcomes during pregnancy and breastfeeding has been reported,^[^ ^71^ ^]^ more data is needed. In the pilot trial of anakinra for endometriosis treatment, no impact was seen on the menstrual cycle, a desirable attribute for patients seeking fertility, and patients' quality of life was significantly improved by anakinra. Larger trials with detailed analysis of excised tissues will be required to dissect the mechanistic underpinnings of the beneficial effects of anakinra for endometriosis, but a clear window of opportunity exists for the control of the pervasive pathologic inflammation in this disease.^[^ ^72^ ^]^ Convergence of evidence highlighting IL1A/B as a central hub of pro‐inflammatory signaling in endometriosis, together with promising early clinical data^[^ ^59^ ^]^ supports continued investigation of IL1R therapy for endometriosis, where new non‐hormonal therapeutic options are urgently needed.

## 4 Experimental Section

### Single Cell Endometriosis Profiles, Immune Cell Annotation

Data from GSE213216 were used for analysis.^[^ ^15^ ^]^ 101217 T/natural killer T‐cells, 27436 myeloid cells, 8278 B/plasma cells, and 1687 mast cells were included in the analysis, plus 10179 additional cells that originally clustered with epithelial and mesenchymal cells but were removed from analysis due to the absence of epithelial or mesenchymal cell markers and positive expression of canonical immune markers. Four samples annotated as “endometriosis‐free” in our initial report^[^ ^15^ ^]^ were reclassified as peritoneal endometriosis due to the detection of endometriosis‐type epithelium and/or stroma in those specimens. CD45 (*PTPRC*) plus additional immune markers were used to move cluster 23, 74, and 101 (cluster number from Fonseca et al,^[^ ^15^ ^]^ these clusters were originally subgrouped with mesenchymal cells but excluded during reclustering) to the T/NK cell group. Any other clusters that express immune markers but low expression of *PTPRC* (ie, certain myeloid) were also selected to be moved (cluster 103 to myeloid group). From the cells removed from the epithelial group, cluster 53 was moved to the myeloid cell group. All immune cell clusters (B/plasma cells, mast cells, myeloid cells, T/NK cell clusters) were reclustered at resolution 3. Clusters that did not express any immune cell markers were removed. The remaining clusters were reclustered at a resolution of 0.6, resulting in 24 clusters. Immune clusters were annotated using canonical markers for T cells (*CD3D*, *CD8A*, *CD4*; 87953 cells), natural killer cells (*KIR2DL4*, *NCAM1*; 5977 cells), myeloid cells (*CD14*, *LYZ*, *CD68*; 15483 cells), B cells, plasmablast/plasma cells (*MS4A1*, *CD79A*, *JCHAIN*, *SDC1*, *CD27*, 5274 and 1771 cells) and mast cells (*KIT*, *TPSB2*; 1645 cells). Seurat clusters expressing markers for the same cell type were merged, such as 5 clusters with markers for Effector Memory CD8 T‐cells are all labeled the same, resulting in 15 distinct functional populations. Unless otherwise specified, analysis was performed using R programming language 4.3. Figures were ordered on Inkscape and icons taken from Bioicons.

### scDRS Analysis

GWAS summary statistics were downloaded for endometriosis and 16 other traits.^[^ ^73^, ^74^, ^75^, ^76^, ^77^, ^78^, ^79^, ^80^, ^81^ ^]^ Trait sample size and ID are listed in Table S6 (Supporting Information). Sample size was maintained over 10 0000 to follow software recommendations, except for arthritis, which only includes 97173 subjects. When missing, reference SNP identifiers were obtained using intersect bedtools between GWAS coordinates and all GRCh37 SNPs. Reference SNP identifiers are needed by MAGMA^[^ ^82^ ^]^ to get a score of disease association per gene. The scDRS software takes as input MAGMA scores for the top 1000 associated genes and outputs a score and *p*‐value per cell. Afterwards, group analysis was tested with the downstream command of the scDRS software for cell type, disease class, and stage. rASRM stages 1 and 2 are relabeled as low stage, while 3 and 4 become high. All scDRS steps were run with default parameters, without including covariates.

### Statistical Analysis—Genomics

Linear regressions linking the score with cell type and stage were fit to test the variance explained by each variable. Spearman correlation was estimated between the expression pattern of every gene and the disease risk score. Correlation was used for ranking and GSEA functional enrichment against the database of Reactome pathways. Pathway activity was estimated using the progeny R package.^[^ ^22^ ^]^ Activity estimates were binned and contrasted with the scDRS, per cell type.

### eQTL Mapping

FUMA v1.6.1^[^ ^83^ ^]^ was used to map cis‐eQTLs to endometriosis GWAS variants in the chromosome 2q14.1 locus using a GWAS *p*‐value threshold of *p *< 5 × 10^−8^ to select SNPs and FDR<0.05 to select SNP‐Gene associations. The locus was defined as ±1 MB from the lead GWAS SNPs (rs3783513 & rs10167914). Similarly, to investigate if GWAS variants in the region were associated with regulation of expression in immune cell types, significant (*p* < 5 × 10^−8^) GWAS variants were mapped in the chromosome 2q14.1 locus to cis‐eQTLs identified using scRNA‐seq in the ONEK1K study^[^ ^84^ ^]^ consisting of 26597 independent eQTLs (*p *< 0.05) across 14 immune cell types.^[^ ^84^ ^]^ The most significant eQTL for each gene in each tissue/cell type was plotted.

### Analysis of Bulk RNA Sequencing Data From Isolated Epithelial and Stromal Cells

Using freshly isolated endometrial epithelial and stromal cells from women with and without endometriosis and performing bulk RNA‐sequencing analysis, the expression of IL1 signaling markers were assessed, which included *IL1A, IL1B, IL1R1, IL1R2, IL1RAP, MYD88, IRAK1, IRAK4, TRAF6, IKBKB, IKBKG, NFKB1*, and *NFKB2*. Markers are taken from Reactome pathway R‐HSA‐9020702. Differential gene expression analysis was performed after TMM normalization, with a linear regression model and included cell type, cycle stage at isolation, and presence of endometriosis as covariates in the model.

### In Vitro Modeling of Endometrium and Endometriosis

Following established protocols,^[^ ^85^, ^86^, ^87^ ^]^ complex in vitro models of endometrium were established to assess IL1/IL1R expression in mature, refluxed endometrium by generating epithelial organoids from menstrual fluid and combining with endometrial stromal cells to introduce impacts derived from cellular interaction. The impact of hormonal exposure on endometrial maturation was also assessed in vitro by initiating organoid models with endometrial stem cells and exogenously mimicking hormonally stimulated endometrial development through the proliferative, secretory, and subsequent “ectopic” stage.

Human menstrual fluid was collected from premenopausal women using a silicone menstrual cup (Lunette, Juupajoki, Finland) as described previously.^[^ ^87^ ^]^ Informed consent was obtained for each patient before surgery. Epithelial cell adhesion molecule (EPCAM) magnetic beads (CELLection Epithelial Enrichment kit, Invitrogen) were used to enrich epithelial cells that were subsequently seeded into Matrigel (7000 clusters per well) and maintained in organoid‐specific media.^[^ ^88^ ^]^ To generate allogeneic organoid co‐cultures endometrial stromal cells were obtained from endometrial biopsies from 8 women (4 with and 4 without endometriosis), separated and cultured as described previously^[^ ^86^ ^]^ and subsequently mixed at a ratio of 1:1 of organoid to stromal cell, with a subsequent ratio of 1:7 of cell mixture with Matrigel (Corning, Matrigel Matrix, GFR and PhenolRed Free, Merck, Australia) to establish mature endometrial in vitro models.

To generate hormonally stimulated endometrial epithelial organoid‐stromal co‐cultures, endometrial biopsies were dissociated to isolate single epithelial progenitor cells and mesenchymal stem cells. Endometrial mesenchymal stem cells were collected from the mesenchymal compartment and induced into a stem cell state through seeding at a sparse density (5000 cells cm^−2^) to achieve clonal growth from individual cells and maintained (> 1 month) in specialized stem cell media (DMEM/F12, 1 µm A83‐01, 10 ng mL^−1^ EGF and 10 ng mL^−1^ FGF2). Media components for organoid growth were based on previously published papers.^[^ ^87^, ^89^ ^]^ Mesenchymal stem cell capacity was confirmed through the ability to differentiate cells into alternate mesodermal lineages of osteoblasts, chondrocytes, and adipose cells. Single epithelial progenitors and mesenchymal stem cells were combined in a 1:1 ratio, seeded into Matrigel, and maintained in organoid media, allowing co‐ordinate maturation of both epithelial and mesenchymal cells.^[^ ^90^, ^91^ ^]^ To simulate hormone exposure and menstrual stage cycles immature organoid‐stromal co‐cultures were exposed to 10 nm 17Beta‐Estradiol, (Merck, Australia) (day 0–5; proliferative stage), with a subsequent 10 nm estrogen, 1 µm progesterone (Merck, Australia) and 1 µm cAMP (Merck, Australia)^[^ ^87^, ^92^ ^]^ (day 5–12; secretory stage). To mimic exposure to an inflammatory ectopic environment, hormonally‐matured organoids were exposed to 10 ng µL^−1^ IL1B (Merck, Australia) (day 12–19). Cell growth and morphology were monitored via growth in the Incucyte Imaging system (Sartorius).

### Statistical Analysis—Organoid Single Cell Transcriptome Data

Single‐cell RNA‐sequencing of the organoid‐stromal co‐cultures produced transcriptomes for 39568 endometrial epithelial cells. The default Seurat pipeline for dataset integration was run to transfer labels from the patient data to the organoids dataset, including SCT normalizing and anchor finding based on variable genes. The 2.81% of cells with a prediction score below 0.75 were discarded as non‐identifiable. The quality of the results was assessed based on the expression of published cell type markers. IL1 signaling was evaluated through the expression of the pathway components and a T‐test contrast.

### In Vivo Assays—Induction of Endometriosis‐Like Lesions

Lesions were induced as described previously.^[^ ^33^ ^]^ Briefly, after at least one week of acclimatization, donor mice received a subcutaneous injection of 3 µg/mouse estradiol benzoate to stimulate the growth of the endometrium. Four days later, the uteri of the donor mice were dissected into a Petri dish containing Hank's Balanced Salt Solution (HBSS, Thermo Fisher Scientific, Waltham, MA, USA) and split longitudinally with a pair of scissors. Uterine horns from each donor mouse were minced with scissors and a scalpel one at a time, ensuring that the maximal diameter of each fragment was consistently smaller than 1 mm (millimeter). Each dissociated uterine horn was then injected intraperitoneally using an 18G needle (cat #305185 Thin wall, BD, Franklin Lakes, NJ, USA) into a recipient mouse in 500 µL of HBSS. One donor mouse was used for every two endometriosis mice.

### Study Design

Block randomization was used to randomize subjects into groups of 10 mice each. Experiments were powered for determining responses to pain but not lesion size. Mice were treated by injection with anakinra (Boston Children's Hospital Pharmacy) in sterile saline (0, 25 mg kg^−1^ day^−1^; 0, 10, 30, 100 mg kg^−1^ day^−1^) starting at day 29 and ending on day 56. The investigators were blinded to the treatment groups in all testing until the end of the experiment and analysis.

### Behavioral Testing

Mice were allowed to habituate to the apparatus for at least 2 h and during three consecutive days before the beginning of measurements. After habituation, baseline measurements were obtained one day before the induction of endometriosis. Pain intensity to a mechanical stimulus (mechanical hyperalgesia) in the abdominal region was measured using von Frey filaments. The experimenter was trained, and care was taken not to stimulate the same point consecutively, and the stimulation of the external genitalia was avoided. A jump or paw flinch was considered a withdrawal response.^[^ ^93^ ^]^ The mechanical threshold was determined by the up and down method, starting with 0.4 g filament and calculated using the open‐source software Up‐Down Reader.^[^ ^94^ ^]^

For spontaneous abdominal pain measurements, stretching the abdomen (abdominal contortions), and squashing of the lower abdomen against the floor were quantified as previously described.^[^ ^93^, ^95^, ^96^ ^]^ For abdominal contortions, mice were placed in individual chambers in a temperature‐controlled (29 °C) glass plate, and the number of abdominal contortions was quantified with a positive response consisting of a contraction of the abdominal muscle together with stretching of hind limbs. For abdominal squashing, the number of times the mice pressed the lower abdominal region against the floor was determined. In all testing, the investigators were blinded to the treatments.

### Statistical Analysis—Murine Experiments

Results are presented as mean ± SEM. Data were analyzed using the software GraphPad Prism version 10.1.2 (GraphPad Software, San Diego, CA, USA). Two‐way repeated measure analysis of variance (ANOVA), followed by Tukey's *post hoc*, was used to analyze data from mechanical hyperalgesia. An unpaired T‐test was used to analyze data from experiments with a single time point. For the percentage of mice with visible lesions, statistical analysis was estimated by the Kaplan‐Meier method followed by the log‐rank test. For all analyses, statistical differences were considered significant when *p* < 0.05.

### Angiogenesis Array and VEGF ELISA

Lesions were dissected at 56 dpi and snap frozen. They were then thawed into lysis buffer (PBS, 1% Triton X‐100) with Roche cOmplete protease inhibitor cocktail (Millipore Sigma, Burlington, MA, USA; catalogue number 4693116001), homogenized (Bio‐Gen model PRO200, PRO Scientific, Oxford, CT, USA), and centrifuged (10,000 g × 5 min, 4 °C). The resulting supernatants were used in a mouse VEGF ELISA array (R&D Systems, Minneapolis, MN, USA, catalogue numberMMV00) or to determine protein levels using the Pierce BCA Protein Assay Kit (Life Technologies, ThermoFisher Scientific, Waltham, MA, USA) followed by the angiogenesis array (R&D Systems, catalogue number ARY015). The angiogenesis assay was conducted with 600 µg of protein for each membrane. One membrane was used for each group, and each membrane corresponded to the protein levels of a pool of five lesions per group. The analysis of each membrane was conducted by densitometry as previously described.^[^ ^97^ ^]^

### Ethics Statement

All mouse work was performed according to protocols approved by the Institutional Animal Care and Use Committee (IACUC) at Boston Children's Hospital (protocols 19‐12‐4054R and 00001816). The work followed the Guide for the Care and Use of Laboratory Animals and all of the regulatory protocols set forth by the Boston Children's Hospital Animal Resources at Children's Hospital (ARCH) facility.

Work with patient‐derived samples was approved by the local IRB, Monash Health HREC, and the US Department of Defence OHRO (RES‐19‐0000‐388A/E00700.1a.1b) and by the University of Queensland Human Research Ethics Committee (2016001723).

## Conflict of Interest

The authors declare no conflict of interest.

## Author Contributions

S.O., F.S.R.‐O. and B.M.K. contributed equally to this work. S.O., K.L., G.W.M., M.S.R., and B.M. performed conceptualization; S.O., F.S.R.‐O, S.M., S.C., and P.Y. contributed to formal analysis and investigation; MH performed data curation; S.S. and C.E.G. acquired resources; S.O. and K.L. wrote the original draft; All authors wrote, reviewed and edited the final manuscript; K.L., G.W.M, and M.S.R. supervised the project; K.L., M.S.A., G.W.M., C.E.G., and M.S.R. acquired funding.

## Code Availability

The code used for figure generation, scDRS and CellChat analysis is provided in: https://github.com/lawrenson‐lab/scDRS_endometriosis

## Supporting information



Supporting Information

Supporting Information

Supporting Information

Supporting Information

Supporting Information

Supporting Information

## Data Availability

The single cell endometriosis atlas data is publicly available at GSE213216. The rest of the datasets are in the process of submission.
