# Supplementary material for: M2 Macrophages are Major Mediators of Germline Risk of Endometriosis and Explain Pleiotropy With Comorbid Traits
Source: Adv Sci (Weinh). 2025 Sep 12;12(41):e15285. doi: 10.1002/advs.202415285 (PMC12591210; doi:10.1002/advs.202415285)
Supplement: Supplementary file 1 — Supporting Information [file ADVS-12-e15285-s004.docx]

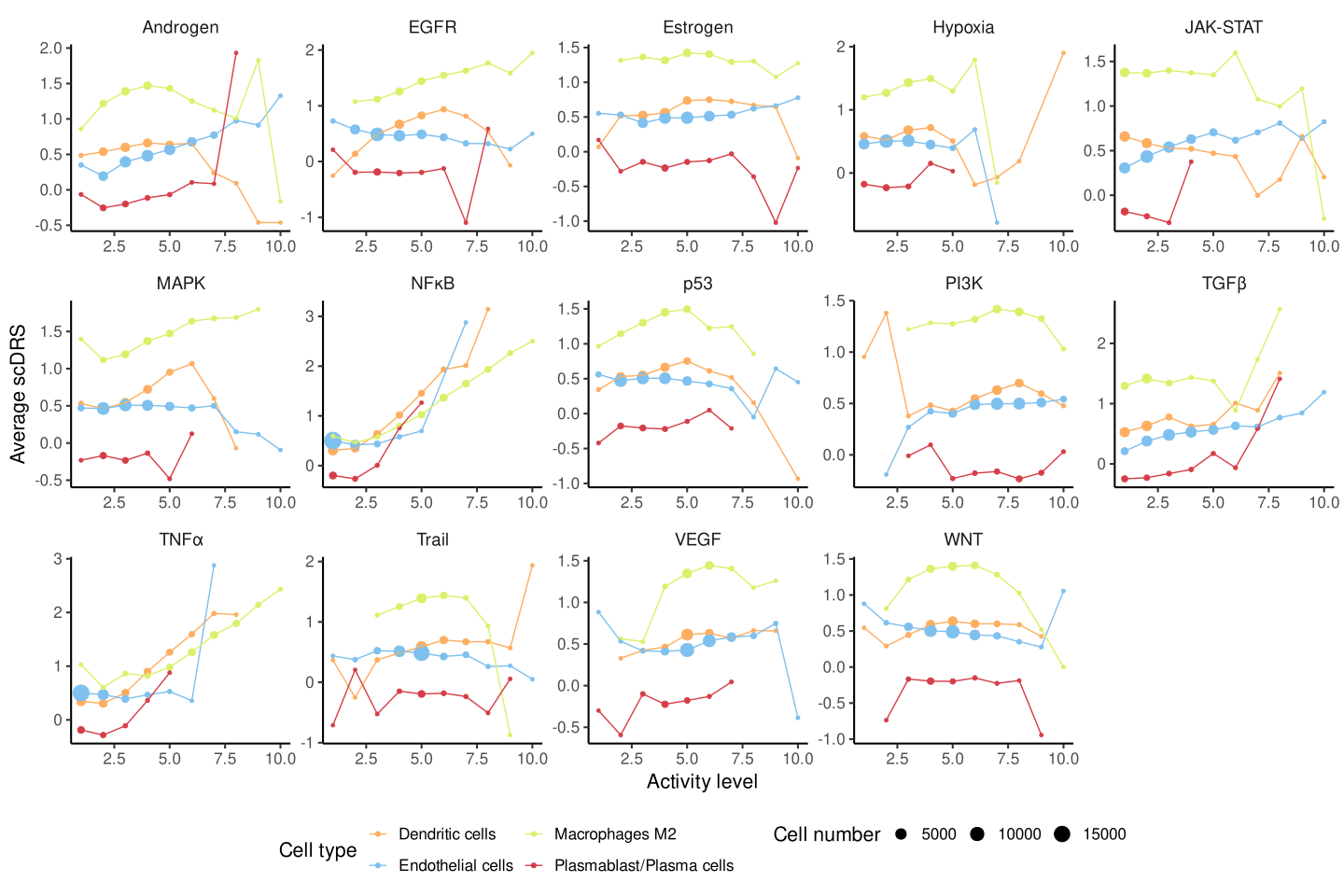


**Supplementary figure 1. scDRS heterogeneity relative to pathway activity.** While most cells have low activity levels for all pathways, matching the expected for single cell data, M2 macrophages cluster around intermediate levels of NFκB and TNF-α.

**
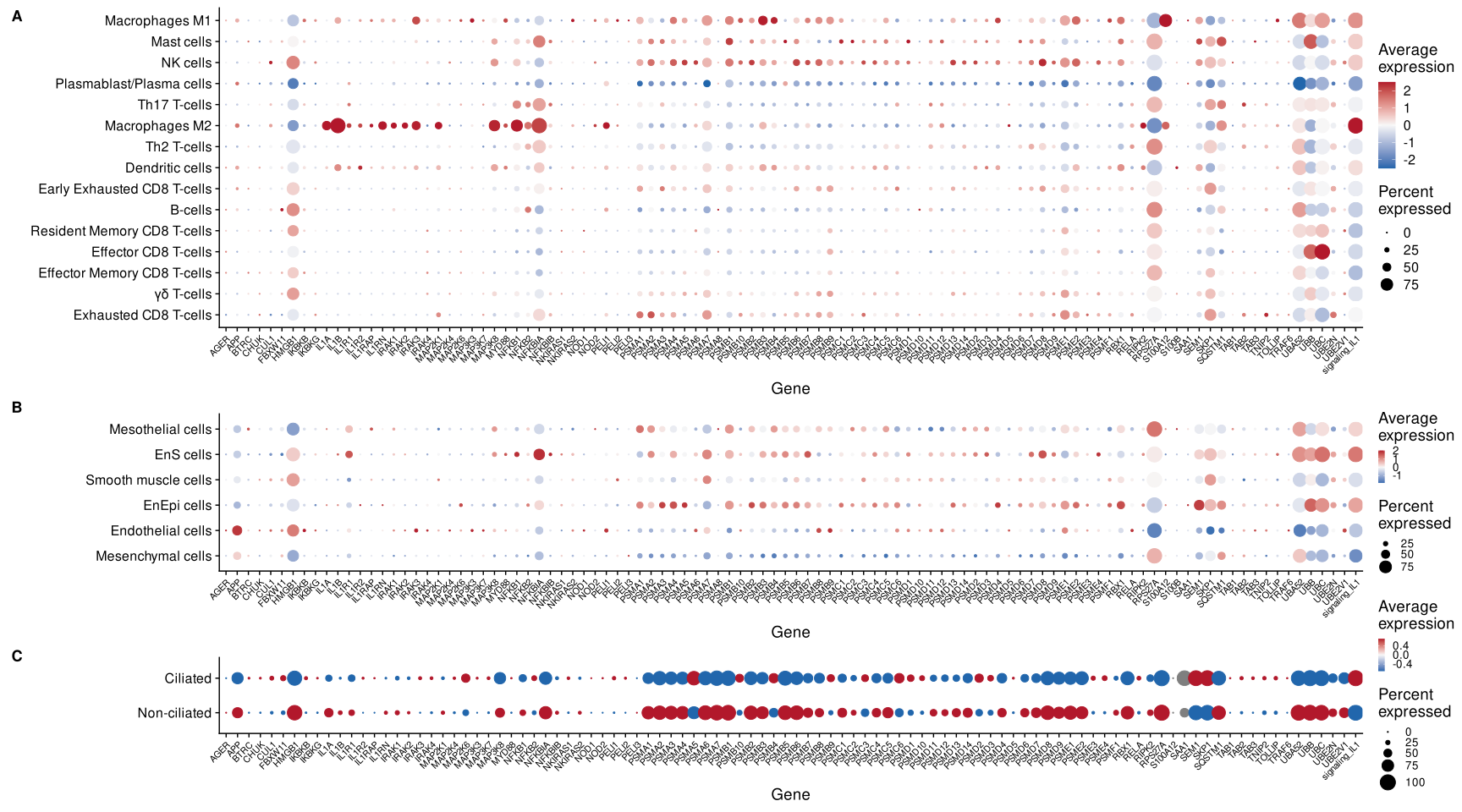
**

**Supplementary figure 2. Expression of the IL1 signature and associated genes per cell cluster. A**) immune subsets from patient single cell data. **B**) non-immune subsets from patient single cell data. **C**) organoid single cell data


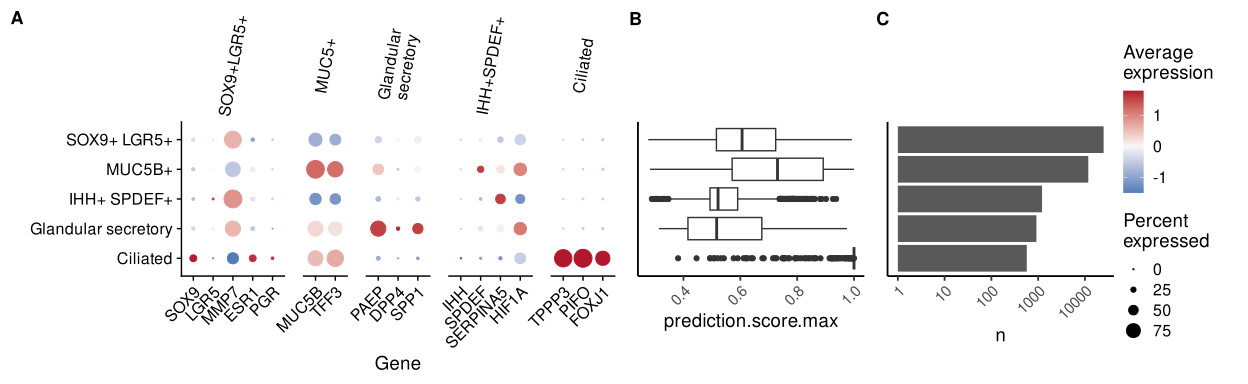


**Supplementary figure 3. Label transfer results for the endometrial epithelial subclusters. A**) Expression of markers from the endometrial epithelial subclusters. **B**) Label assignment score. **C**) Number of cells in each cluster

**
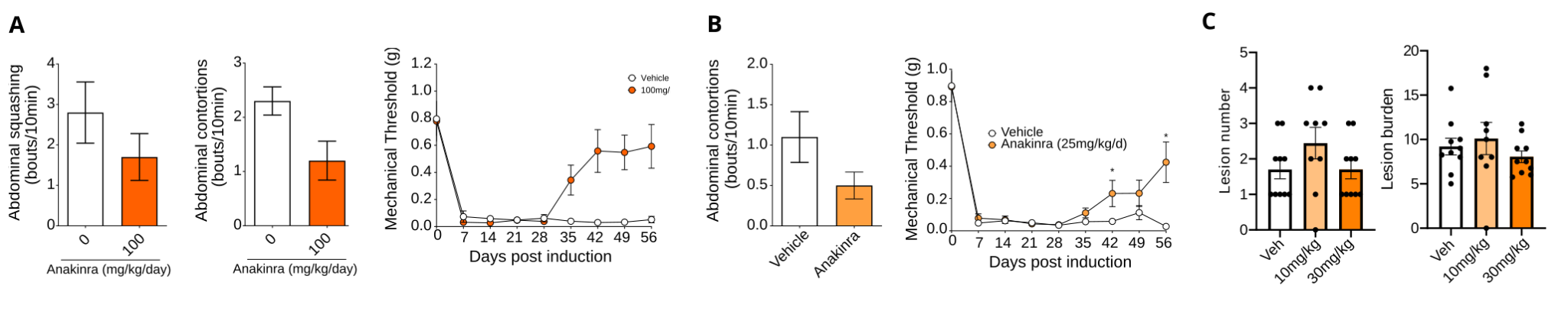
**

**Supplementary figure 4. Additional data from anakinra treatment experiments.** Endometriosis-like lesions were induced in C57BL/6J mice, allowed to grow for 4 weeks, and treated with anakinra at the indicated dose. Spontaneous pain was decreased following 4 weeks of treatment. **A**) Spontaneous and evoked pain in animals treated with 100 mg/kg anakinra beginning on day 29. **B**) Spontaneous and evoked pain in animals treated with 25 mg/kg anakinra beginning on day 29. **C**) Lesion parameters for mice in Figure 5 C-G. N=10 mice per group except for the 10 mg/kg group where N=9.​
