# Supplementary figures and images for: M2 Macrophages are Major Mediators of Germline Risk of Endometriosis and Explain Pleiotropy With Comorbid Traits
Source: Adv Sci (Weinh). 2025 Sep 12;12(41):e15285. doi: 10.1002/advs.202415285 (PMC12591210; doi:10.1002/advs.202415285)

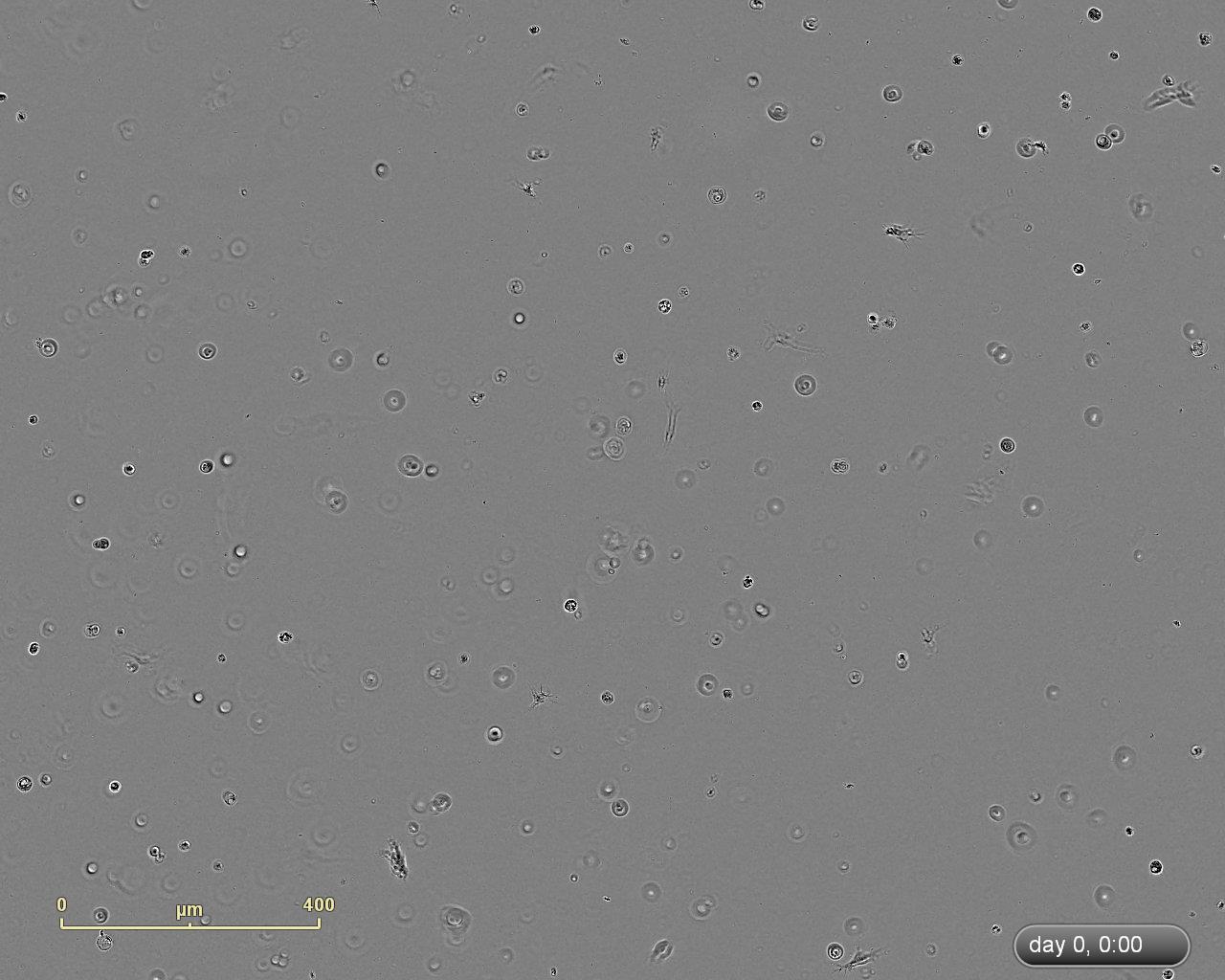

Supplement: Supplementary file 3 — Supporting Information [file ADVS-12-e15285-s005.jpg]

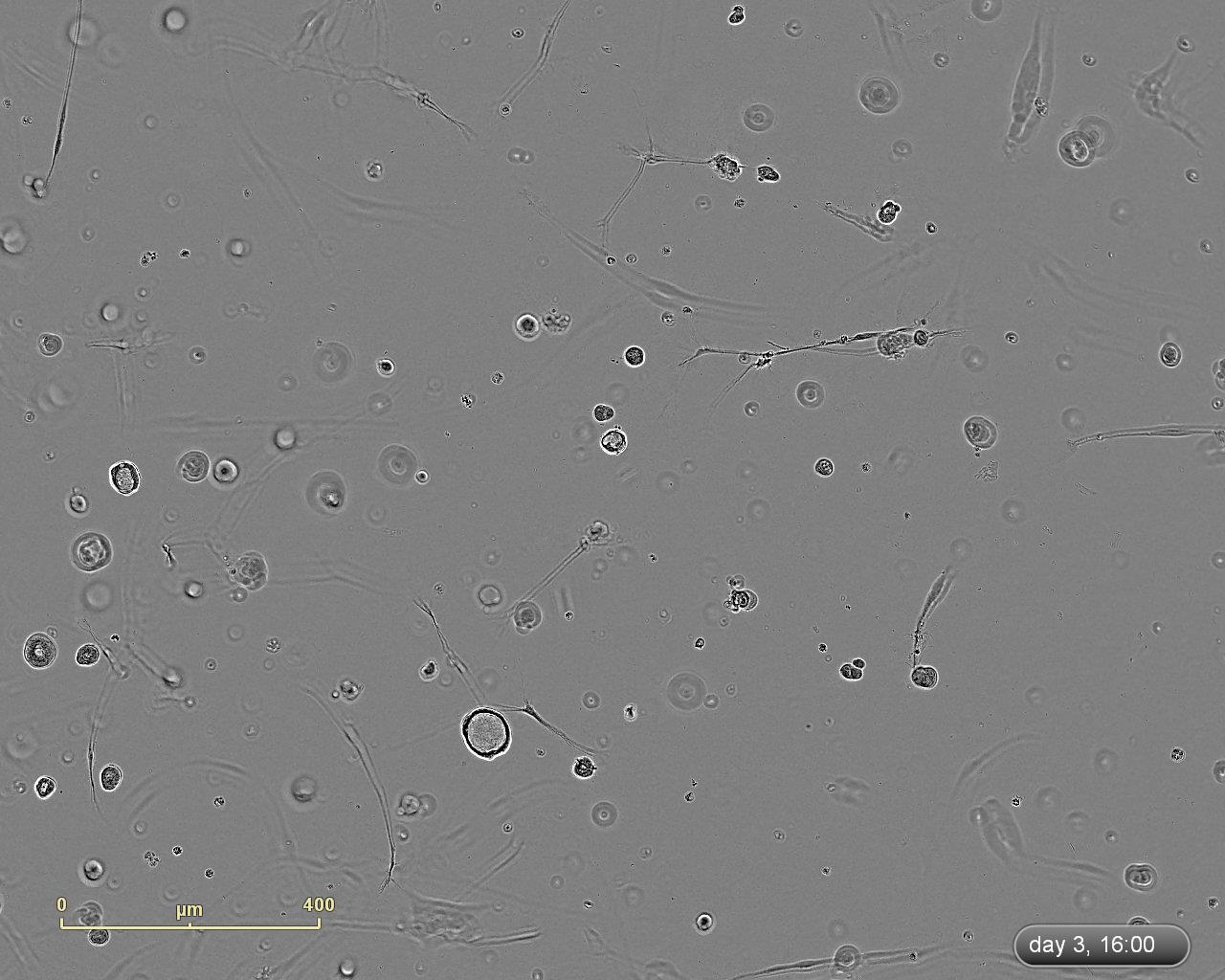

Supplement: Supplementary file 4 — Supporting Information [file ADVS-12-e15285-s002.jpg]

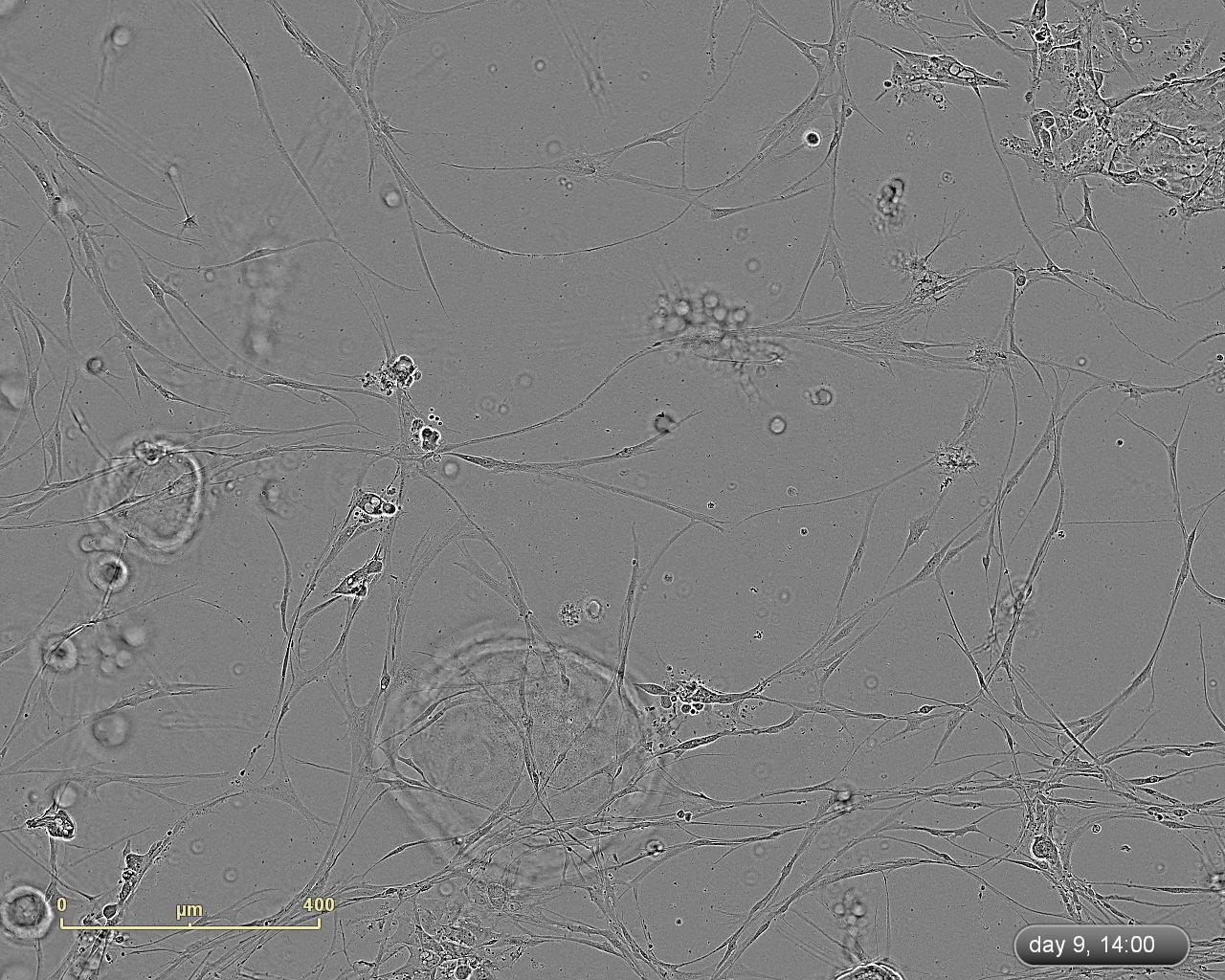

Supplement: Supplementary file 5 — Supporting Information [file ADVS-12-e15285-s001.jpg]

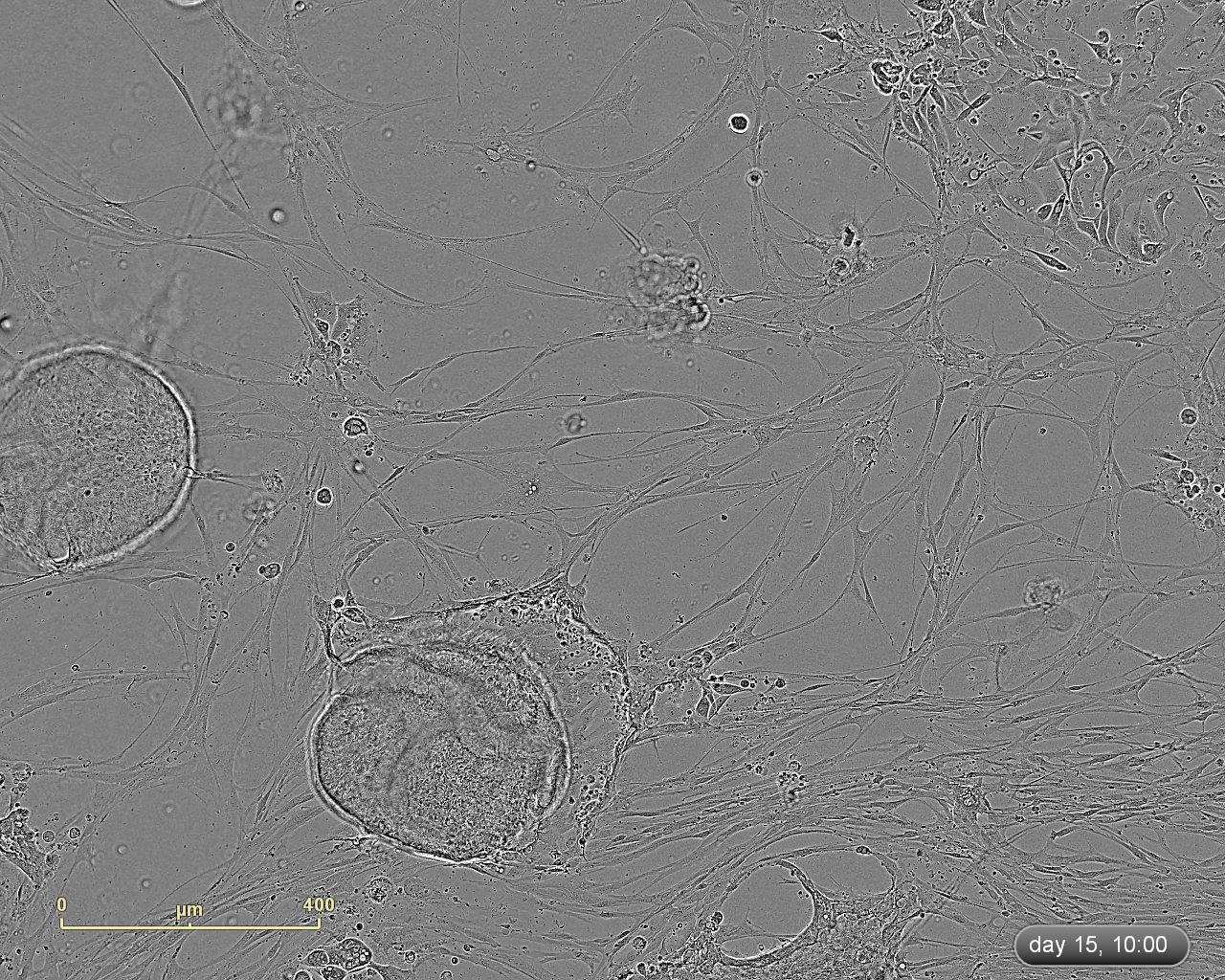

Supplement: Supplementary file 6 — Supporting Information [file ADVS-12-e15285-s006.jpg]
